# YBX1: an RNA/DNA-binding protein that affects disease progression

**DOI:** 10.3389/fonc.2025.1635209

**Published:** 2025-07-29

**Authors:** Xifeng Zheng, Feng Zeng, Yan Lei, Yanling Li, Jun Deng, Gengqiu Luo, Qian He, Yanhong Zhou

**Affiliations:** ^1^ Department of Radiation Oncology, The Affiliated Cancer Hospital of Xiangya School of Medicine Central South University/Hunan Cancer Hospital, Changsha, Hunan, China; ^2^ Cancer Research Institute, Basic School of Medicine, Central South University, Changsha, Hunan, China; ^3^ Department of Blood Transfusion, The Affiliated Cancer Hospital of Xiangya School of Medicine Central South University/Hunan Cancer Hospital, Changsha, Hunan, China; ^4^ Department of Nuclear Medicine, The Affiliated Cancer Hospital of Xiangya School of Medicine, Central South University/Hunan Cancer Hospital, Changsha, Hunan, China; ^5^ Department of Early Clinical Trail Center, The Affiliated Cancer Hospital of Xiangya School of Medicine Central South University/Hunan Cancer Hospital, Changsha, Hunan, China; ^6^ Department of Pathology, Xiangya Hospital, Basic School of Medicine, Central South University, Changsha, Hunan, China

**Keywords:** YBX1, tumor metastasis, chemotherapy resistance, RNA stability, non-coding RNA interactions

## Abstract

YBX1 is a member of the Y-box transcription factor family and is a multifunctional RNA/DNA-binding protein characterized by a highly conserved cold shock domain (CSD). YBX1 is localized in both the cytoplasm and nucleus, where it participates in various biological processes such as transcription, translation, and DNA damage repair. YBX1 is upregulated in numerous malignant tumors and is closely associated with tumor progression and poor prognosis, making it a recognized target for cancer therapy. This review introduces the role and mechanism of YBX1 in tumor progression, its function in embryonic development, bone differentiation, cartilage formation, and adipogenesis, the impact of upstream regulatory factors on its function, as well as the relationship between YBX1 and disease prognosis and treatment outcomes. This review aims to provide a comprehensive perspective on the function of YBX1.

## 1 Introduction

YBX1 (Y box binding protein 1) is a member of the YBX transcription factor family and functions as a multifunctional RNA/DNA binding protein. The name “YBX” is derived from the ability of the YBX protein family to bind to the Y-box sequence on DNA, defined as 5’-CTGATTGG-3’. YBX family proteins exhibit high sequence homology across different species and include three members: YBX1, YBX2, and YBX3. Each member contains the classical cold shock protein (CSD) domain, A/P domain, and a long C-terminal domain (1). The term “cold shock” originates from studies in Escherichia coli, which demonstrated a 210-fold increase in the expression of approximately 13 proteins containing CSD when exposed to cold temperatures, aiding in cellular survival under such conditions. This bacterial finding mirrors the role of YBX1 in the stress response of eukaryotic cells, indicating that the YBX family’s structural and functional properties have been preserved across a wide evolutionary span (2).

YBX1 is closely related to the occurrence and development of various diseases, including malignant tumors. It is a recognized oncogenic transcription factor that regulates cell apoptosis, translation, proliferation, mRNA splicing, repair, differentiation, and stress responses (1). Additionally, YBX1 is essential for sorting selected miRNAs into exosomes and plays a significant role in sorting highly abundant small ncRNA species, including tRNA, Y RNA, and Vault RNA (1). YBX1 also influences the transcriptional activity of the 5’ and 3’ promoter elements of HTLV-1 (2). YBX2 is primarily expressed in germ cells and is involved in maintaining the stability of germ cell mRNA (3). In contrast, YBX3 is expressed during embryonic development but is absent in adult cells, where it acts as a repressor of several growth factor promoters (4). YBX1, YBX2, and YBX3 are crucial throughout the cell life cycle, possessing both DNA and RNA binding functions. This review mainly explores the mechanisms by which YBX1 promotes tumor progression, including its roles in embryonic development, bone differentiation, chondrogenesis, and adipogenesis. It also examines the effects of upstream regulators on YBX1 function and the relationship between YBX1 and disease prognosis. This paper aims to provide a new perspective on the comprehensive understanding of YBX1 functions.

## 2 YBX1 promotes tumor progression and its related mechanisms

### 2.1 YBX1 promotes tumor progression through its transcription factor activity

Currently, there is substantial evidence indicating that YBX1 promotes tumor progression through its transcription factor activity. For instance, in lung adenocarcinoma, Xie et al. confirmed through experiments that MUC1 is a downstream target of YBX1. YBX1 binds to the MUC1 promoter region (-1480bp to -1476bp) to regulate its transcription, suggesting that YBX1 promotes the occurrence and metastasis of lung adenocarcinoma via MUC1 targeting (5). Furthermore, Zhao and colleagues demonstrated that endogenous YBX1 binds to the CDC25a promoter regions, leading to increased CDC25a promoter luciferase expression in human lung adenocarcinoma cells. This indicates that YBX1 regulates tumor growth through the CDC25a pathway (6). Additionally, YBX1 directly promotes the transcriptional activation of NANOG, a transcription factor, which in turn promotes lung cancer stem cell-like characteristics and metastasis (7). In nasopharyngeal carcinoma, YBX1 promotes the expression of AURKA protein by directly binding to AURKA mRNA, thereby promoting the proliferation and invasiveness of nasopharyngeal carcinoma cells (8). Similarly, in colorectal cancer, YBX1 binds to the promoter and acts as a transcriptional activator of the EGFR gene, mediating resistance to anti-ERBB2 therapy and preventing apoptosis in ERBB2-overexpressing breast cancer cells through a complex RSK-dependent mechanism (9). Moreover, in bladder cancer, YBX1 promotes glycolysis by up-regulating the expression of glycolytic enzymes, glucose uptake, lactate secretion, and the extracellular acidification rate (ECAR), thereby promoting tumor growth (10). In osteosarcoma, YBX1 promotes disease progression by up-regulating VEGF165 and down-regulating VEGF165b (11). In pancreatic cancer, YBX1 binds to the GSK3B promoter and promotes the growth of pancreatic ductal adenocarcinoma through the GSK3B/Cyclin D1/Cyclin E1 pathway (12). Lastly, in breast cancer, YBX1 gene silencing reduces the expression of CORO1C gene and inhibits the migration and invasion potential of breast cancer cells (13).

In summary, YBX1 promotes tumor progression through its transcription factor activity, exerting its effects via various downstream targets such as MUC1, CDC25a, NANOG, Kindlin-2, G3BP1, AURKA, SPP1, the EGFR-RAS-MAPK axis, CORO1C, among others, depending on the specific tumor type. While current research has established a strong correlation between the activity of YBX1 as a transcription factor and the progression of various malignant tumors, the precise mechanisms by which YBX1 operates in each tumor context remain incompletely understood. To leverage the targeting of YBX1 transcription factor activity as a molecular therapeutic approach for clinical tumor treatment, further studies are warranted to elucidate the intricate details of its mechanisms across different tumor types.

### 2.2 YBX1 promotes tumor progression by influencing the functions of its interacting proteins

**Figure 1:**
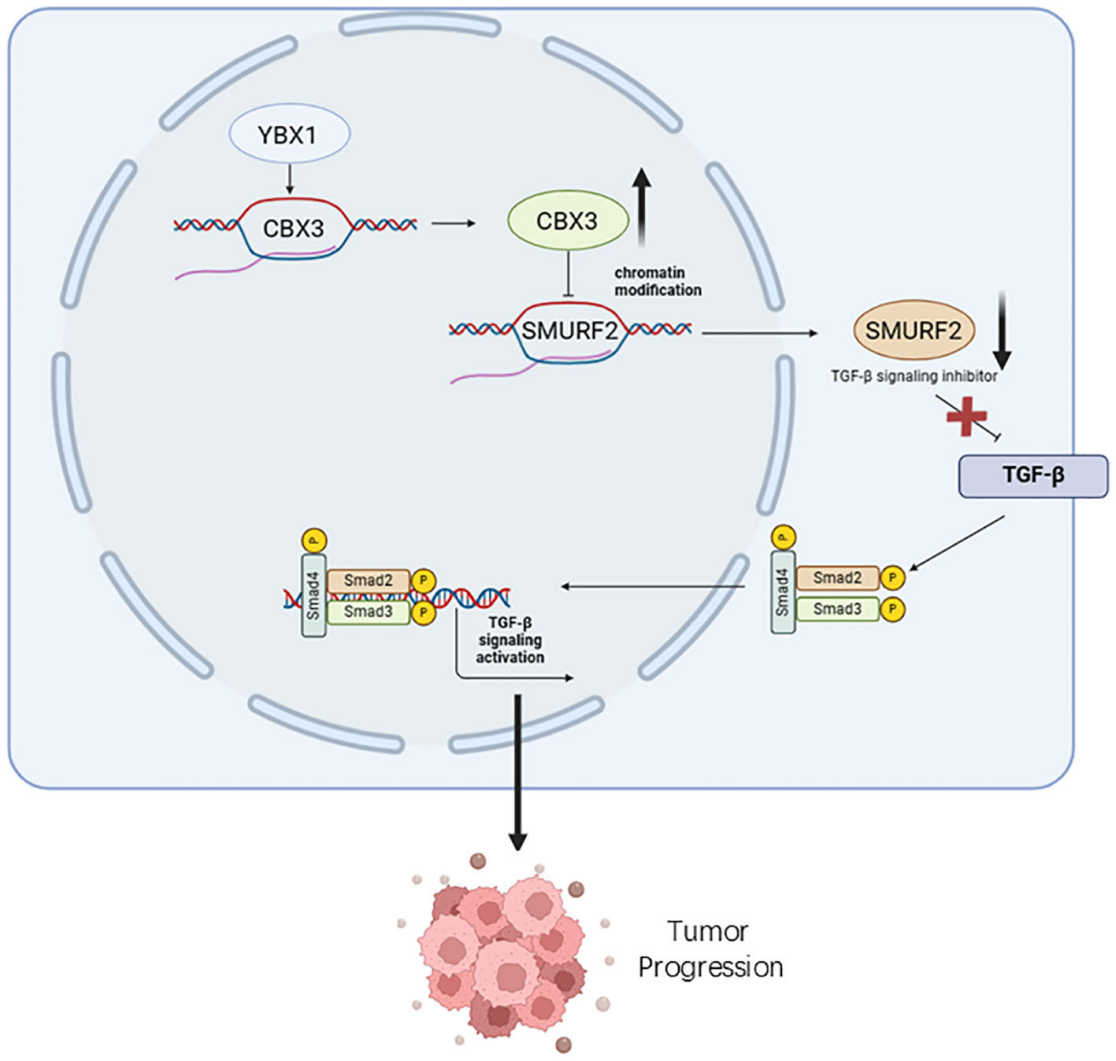
YBX1 promotes tumor progression by influencing the function of its interacting molecules. The overexpression of YBX1 results in the upregulation of CBX3 in cancer cells, leading to the inhibition of SMURF2 expression. Decreased SMURF2 expression enhances TGF-β signaling activation, phosphorylating smad2, smad3, and smad4, thereby promoting pancreatic cancer progression.

In addition to its role as a transcription factor in tumors, YBX1 interacts with downstream factors to further promote tumor progression. For instance, in renal cell carcinoma (RCC), Cui et al. confirmed that YBX1 interacts with Kindlin-2, regulating RCC cell apoptosis and reactive oxygen species (ROS) production by activating Wnt signaling to induce epithelial-to-mesenchymal transition (EMT). This suggests that YBX1 gene silencing induces RCC cell apoptosis through Kindlin-2 (14). In bone cancer, YBX1 stabilizes pro-metastatic mRNA by recognizing sequence motifs shared by the 3’ end of tRNA fragments(tRF)-Gly^TCC^ and RUNX2, thereby inhibiting tumor metastasis in primary bone cancer progression (15). In pancreatic cancer, YBX1 interacts with the epigenetic regulator CBX3 to promote cancer progression by inhibiting the expression of SMAD specific E3 ubiquitin protein ligase 2 (SMURF2) and activating TGF-β signaling (16) (**Figure 1**). In rhabdomyosarcoma, MYC and YBX1 regulate each other; MYC binds to the YBX1 promoter, and YBX1 binds to MYC mRNA, forming a MYC-YBX1 circuit that maintains stem cell-like vincristine-resistant cells (17). In acute myeloid leukemia (AML), YBX1 serves as a disease-sustaining mediator of jak2-mutated myeloproliferative neoplasms, enhancing the translational capacity of oncogenic transcripts (including MYC) by recruiting polysome chains, thereby driving the survival and proliferation of AML cells (18). In glioblastoma, YBX1 is co-expressed with six interacting proteins involved in the cell invasion network, and as a regulator of these key molecules, it participates in regulating the protein network related to tumor invasion (19). In lung cancer, methylated YBX1 interacts with the tumor suppressor hY4F to mediate the selective sorting and secretion of hY4 RNA fragments into extracellular vesicles, leading to up-regulation of MAPK/NF-κB signaling pathway activity and promoting the proliferation, migration, and invasion of lung cancer cells (20). In liver cancer, YBX1 interacts with CBX8 to regulate the cell cycle and promote the proliferation of liver cancer cells (21). In breast cancer, YBX1 and the transcription factor NFIB bind to estrogen receptors and regulate the proliferation, growth, metastasis, and response to hormone therapy of tumor cells through the FGFR2 signaling pathway (22). In esophageal squamous cell carcinoma (ESCC), YBX1-mediated linc02042 and c-MYC form a new positive feedback loop, enhancing the stability and translation efficiency of c-MYC mRNA and promoting tumorigenesis and metastasis (23).

In conclusion, molecules such as RUNX2, CBX3, and MYC interact with YBX1 to promote tumor progression in various malignant tumors, including bone cancer, pancreatic cancer, and rhabdomyosarcoma, among others. However, the regulatory effects of YBX1 and its interacting molecules, as well as the detailed mechanisms of these interactions on tumor progression, require further investigation. Further research in this area is essential for better understanding the complex molecular pathways involved in tumorigenesis and for the development of more effective therapeutic strategies targeting YBX1 and its interacting partners.

### 2.3 YBX1 promotes tumor progression through m^6^A modification, m^5^C modification and other pathways

YBX1 exhibits diverse mechanisms in promoting tumor progression, including its regulation of downstream factors through m6A modification and the PI3K/AKT signaling pathway. For instance, YBX1 interacts with insulin-like growth factor 2 messenger RNA (mRNA)-binding proteins (IGF2BPs)and stabilizes m^6^A-labeled RNA to sustain the survival of myeloid leukemia cells by regulating BCL2 (24). The expression of YBX1 is significantly increased in the hematopoietic stem cells of chronic myeloid leukemia. YBX1 interacts with RNA m^6^A reader IGF2BPs and stabilizes m^6^A-labeled RNA to stabilize the transcription of YWHAZ, thus regulating the survival of CML stem cells (25).

**Figure 2:**
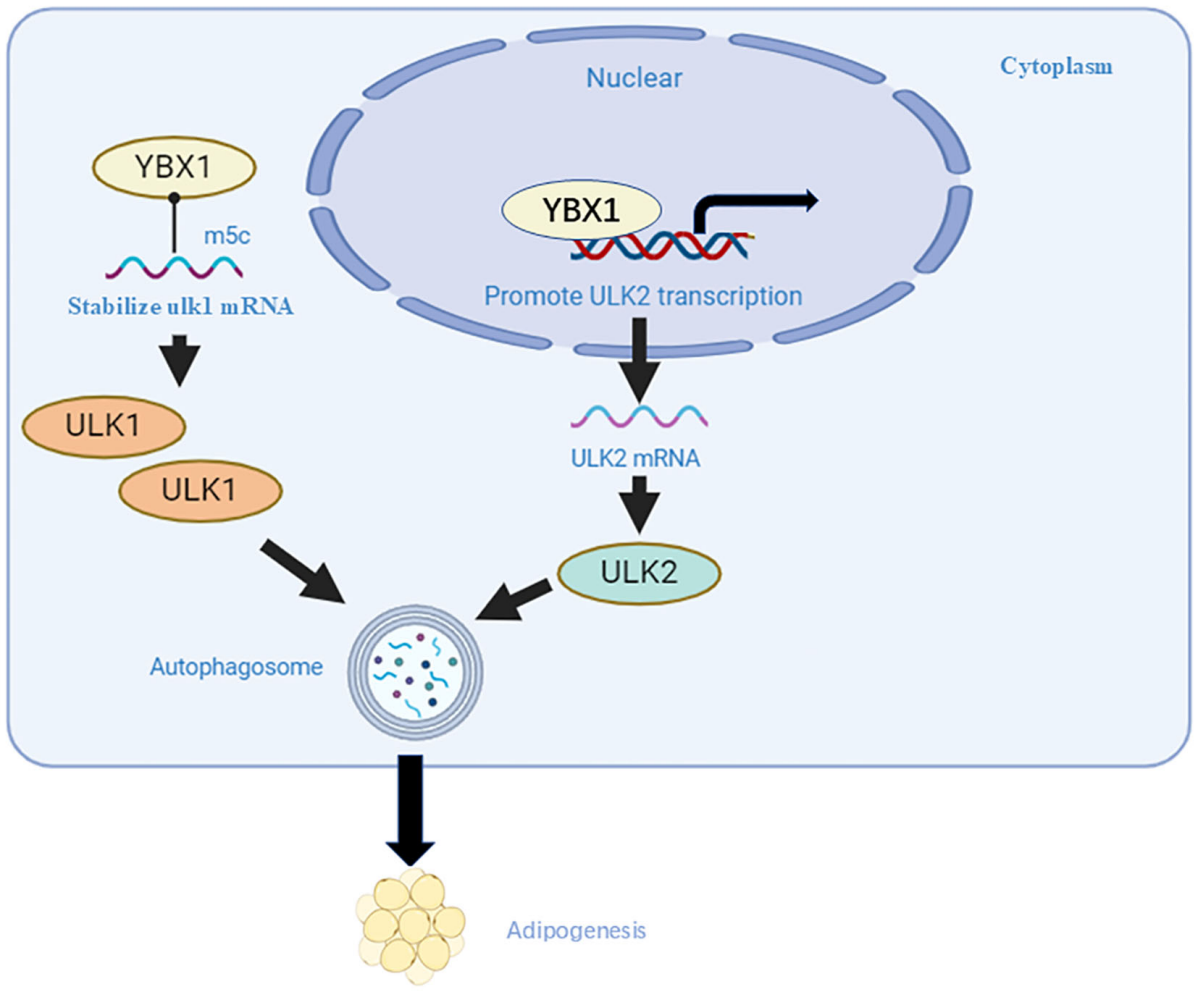
YBX1 positively regulates adipogenesis. YBX1 binds specifically to the Ulk1 transcript containing m5C, stabilizing its mRNA as an RNA-binding protein. Additionally, YBX1 acts as a DNA-binding protein, promoting Ulk2 transcription, consequently increasing Ulk1 and Ulk2 protein levels and enhancing autophagy and adipogenesis.

YBX1 has been identified as a reader of m^5^C modifications. The NSUN2-m^5^C-YBX1 axis represents a well-defined mechanism by which YBX1 recognizes and binds to m^5^C-modified RNAs. This interaction plays a crucial role in regulating mRNA stability, translation, and oncogenic reprogramming. In triple-negative breast cancer, YBX1 in the cytoplasm directly binds to SAT1 protein, and the mTOR mRNA accumulated through YBX1 protein-mediated methyl-5-cytosine (m^5^C) modification is stabilized through E3 ligase HERC5-mediated deubiquitylation, which significantly inhibits autophagy and accelerates tumor progression in triple-negative breast cancer (26). YBX1 has also been identified as an m^5^C binding protein that directly targets m^5^C-containing Ulk1 mRNA, leading to the upregulation of Ulk1 expression by stabilizing its mRNA (**Figure 2**). Additionally, YBX1 acts as a transcription factor to promote the transcription and expression of Ulk2, thereby enhancing autophagy and adipogenesis (27).

Additionally, YBX1 has been implicated in promoting tumor progression through the PI3K/AKT signaling pathway in laryngeal squamous cell carcinoma (LSCC). Its reduced expression leads to decreased activation of PI3K/AKT signaling molecules, promoting LSCC cell apoptosis and suppressing cell proliferation, migration, and invasion (28). Furthermore, YBX1 interacts with lncRNA SBF2-AS1 to modulate cell proliferation via the PI3K/AKT/mTOR signaling pathway, thus influencing the proliferation of breast cancer cells (29). Lastly, in T-cell acute lymphoblastic leukemia (T-ALL), downregulation of YBX1 results in inhibition of total AKT, p-AKT, total extracellular signal-regulated kinase (ERK), and p-ERK expression, playing a pivotal role in the development of T-ALL (30). In conclusion, YBX1 not only promotes tumor progression through its transcription factor activity and interaction with various molecules but also facilitates tumor progression through m6A modification and the PI3K/AKT signaling pathway.

## 3 The effect and mechanism of YBX1 on embryonic development, stem cell differentiation, adipogenesis and other biological behaviors

### 3.1 YBX1 affects embryonic development

YBX1 plays a crucial role in various aspects of embryonic development. For instance, YBX1 homologs, such as YPS in Melanogaster, are essential for the maintenance, proliferation, and differentiation of ovarian germ line stem cells (GSCs) by preferentially binding to m^5^C RNAs. In Drosophila ovaries, YPS functions to maintain GSC proliferation and progeny differentiation, with human YBX1 capable of functionally replacing YPS to support normal GSC development (31). Additionally, in the context of stem cell biology, the interaction of YBX1 and ILF3 with Nanog mRNA positively influences the expression of Nanog and other pluripotency-related genes, contributing to stem cell self-renewal and the maintenance of pluripotent cell identity (32). Furthermore, in zebrafish folliculogenesis, proteomics analysis has identified YBX1 as a key component of messenger ribonucleoprotein particles (mRNPs), suggesting its role as a potential gatekeeping molecule controlling early ovarian folliculogenesis by binding to other RNA-binding proteins (33). Zebrafish maternal YBX1 is associated with the processing of embryonic body components and safeguards zebrafish oocyte maturation and the mother-to-zygote transition by inhibiting global translation when linked to the target mRNA (34). Moreover, long non-coding RNAs in the development of YBX1 gene knockout zebrafish embryos may target reduction oxidation-related genes such as duox (NADPH oxidase) and noxo1a (NADPH oxidase organization), which are involved in the development of zebrafish embryos (35). In summary, YBX1 plays a pivotal role in embryonic development across various species, contributing to processes such as stem cell maintenance, differentiation, and tissue organization.

### 3.2 YBX1 promotes stem cell differentiation

YBX1 plays a significant role in promoting stem cell differentiation, particularly in maintaining the osteogenic potential and anti-aging ability of mesenchymal stem cells (MSCs). For instance, in dental pulp stem cells, YBX1 expression significantly increases the expression of RUNX2 exon 5, thereby promoting the mineralization ability of dental pulp stem cells. Conversely, silencing YBX1 reduces the inclusion of exon 5 and the corresponding RUNX2 protein expression level (36). Moreover, the expression of YBX1 decreases with aging in both mouse and human bone marrow-derived mesenchymal stem cells (BMSCs). YBX1 deficiency leads to aberrant splicing of genes related to osteogenic differentiation and senescence of BMSCs, such as Fn1, Nrp2, Sirt2, Sp7, and Spp1, resulting in BMSC senescence and impaired differentiation and metastasis during aging (37). Additionally, YBX1 levels gradually increase during the osteogenic differentiation of BMSCs, and YBX1 silencing inhibits the PI3K/AKT pathway. Thus, YBX1 promotes the osteogenic differentiation of MSCs by activating the PI3K/AKT pathway (38). These findings underscore the critical role of YBX1 in regulating stem cell fate, highlighting its potential as an important molecular target for promoting stem cell differentiation and tissue regeneration.

### 3.3 YBX1 positively regulates adipogenesis

YBX1 plays a crucial role in adipogenesis, as evidenced by various studies. YBX1 directly targets the transcripts of PTEN-induced kinase 1 (Pink1) and Parkin RBR E3 ubiquitin-protein ligase (Prkn). Deletion of the YBX1 gene reduces the mRNA stability of Pink1 and Prkn, resulting in decreased protein expression, thereby alleviating mitophagy and inhibiting the thermogenic program (39). Moreover, YBX1 is sharply upregulated in brown adipose tissue after cold exposure, induces YBX1 expression in mesenchymal stem cells during adipogenesis, promotes browning, and simultaneously increases the expression of thermogenic markers and enhances mitochondrial respiration. These findings identify YBX1 as a novel factor that coordinates the genomic mechanism of preadipocyte transition to white/beige lineage, thereby positively regulating adipogenesis (40). In summary, YBX1 plays a significant role in promoting adipogenesis through various mechanisms, highlighting its importance in the regulation of adipocyte differentiation and function.

### 3.4 YBX1 affects the development and function of the nervous system

YBX1 plays a crucial role in neurological disorders, influencing the development and function of the nervous system. For instance, YBX1 interacts with the key chromatin modifier PRC2, which is involved in the regulation of embryonic brain development by fine-tuning the activity of PRC2 during neurodevelopmental processes. In mouse embryos, YBX1 is essential for forebrain development and the restriction of midbrain development. Additionally, in neural progenitor cells (NPCs), YBX1 governs self-renewal and neuronal differentiation (41). In the context of ischemic cerebral apoplexy, extracellular vesicle-bound YBX1 from neural stem cells interacts with IGF2BP1. This interaction increases the m6A modification of G protein-coupled receptor 30 (GPR30) stability and expression. Elevated GPR30, mediated by SPOP, promotes NLRP3 ubiquitination, inhibiting the activation of NLRP3 inflammatory bodies and ultimately suppressing neuronal pyroptosis (42). Moreover, YBX1 has been found to bind to the hypermethylation region of the SHANK3 promoter in cortical interneurons derived from induced pluripotent stem cells (iPSCs). This hypermethylation negatively correlates with the cortical surface area of the left inferior temporal cortex (43). In summary, YBX1 plays a crucial role in nervous system development and function, contributing to both embryonic brain development and the regulation of inflammatory responses and epigenetic modifications associated with neurological disorders.

## 4 Effects of the upstream regulators of YBX1 on its function

### 4.1 microRNA

MicroRNAs (miRNAs) are crucial regulators of gene expression, acting post-transcriptionally by binding to specific sequences on target mRNAs. Several miRNAs have been identified as regulators of YBX1 expression through direct targeting of its 3’ untranslated region (3’-UTR), influencing various biological processes. For example, miR-148a-3p directly targets the 3’-UTR of YBX1, leading to reduced expression of YBX1 protein (44). Similarly, miR-216a and miR-137 bind to YBX1 mRNA, promoting its degradation and inhibiting YBX1 expression, thereby impacting cell proliferation, invasion, and chemosensitivity in different cancers (45–47). MiR-376a competitively binds to YBX1 and MEG3, affecting angiogenesis in microvascular endothelial cells (48). MiR-379-5p inhibits the epithelial-mesenchymal transition (EMT) of nasopharyngeal carcinoma cells and the PI3K/Akt pathway in osteoarthritis chondrocytes by targeting YBX1 (49, 50). Additionally, miR-375 regulates YBX1 expression in breast cancer cells, influencing chemotherapy sensitivity and multidrug resistance (51). Furthermore, miR-216a plays a role in diabetic nephropathy by regulating Tsc-22 through YBX1-mediated post-transcriptional regulation (52). In summary, miRNAs exert significant regulatory effects on various biological processes by targeting the 3’-UTR of YBX1 mRNA, highlighting their importance in gene expression regulation and potential therapeutic implications in diseases.

Understanding how miRNAs regulate crucial cellular processes such as proliferation, invasion, migration, and epithelial-mesenchymal transition (EMT) through targeting YBX1 provides valuable insights into the molecular mechanisms underlying these diseases and guides further research directions. However, it is important to note that most studies primarily rely on *in vitro* cell models, which may not fully replicate the complex biological milieu present *in vivo*. Thus, there is a need for more comprehensive *in vivo* experiments to validate these findings and better understand their clinical relevance. Moreover, while miRNAs hold promise as potential therapeutic targets, translating these research findings into clinical applications poses challenges. Issues such as efficient drug delivery, precise dose control, and ensuring safety need to be addressed. Overcoming these challenges will be crucial for harnessing the therapeutic potential of miRNAs in clinical settings and realizing their benefits for patients with various diseases. Continued research efforts in both basic science and clinical translation are essential for advancing our understanding of miRNA-mediated regulation of YBX1 and exploring its therapeutic implications.

### 4.2 lncRNA

Long non-coding RNAs (lncRNAs) are RNA molecules longer than 200 nucleotides that play crucial roles in various biological processes, including dosage compensation, epigenetic regulation, cell cycle regulation, and cell differentiation. Recent studies have revealed that lncRNAs can modulate the function of YBX1 by regulating the miRNA/YBX1 axis. For instance, the long non-coding RNA PRKCQ-AS1 promotes the proliferation and migration of colorectal cancer cells by regulating the miR-1287-5p/YBX1 axis (53). Similarly, long non-coding RNA Linc01612 inhibits the progression of liver cancer by regulating the miR-494/ATF3/p53 axis and promoting YBX1 ubiquitination (54). Additionally, long non-coding RNA PVT1 downregulates miR-216a-5p and inhibits the progression of colorectal cancer by regulating YBX1 expression (55). Furthermore, long non-coding RNA HOXA11-AS targets the miR-337-3p/YBX1 signaling pathway to regulate ischemic neuronal death (56). Lastly, lncNMR targets the YX1-RRM2 axis to regulate nucleotide metabolism in cancer (57). These findings underscore the intricate regulatory networks involving lncRNAs, miRNAs, and YBX1, highlighting the importance of lncRNA-mediated regulation in various physiological and pathological processes.

**Figure 3:**
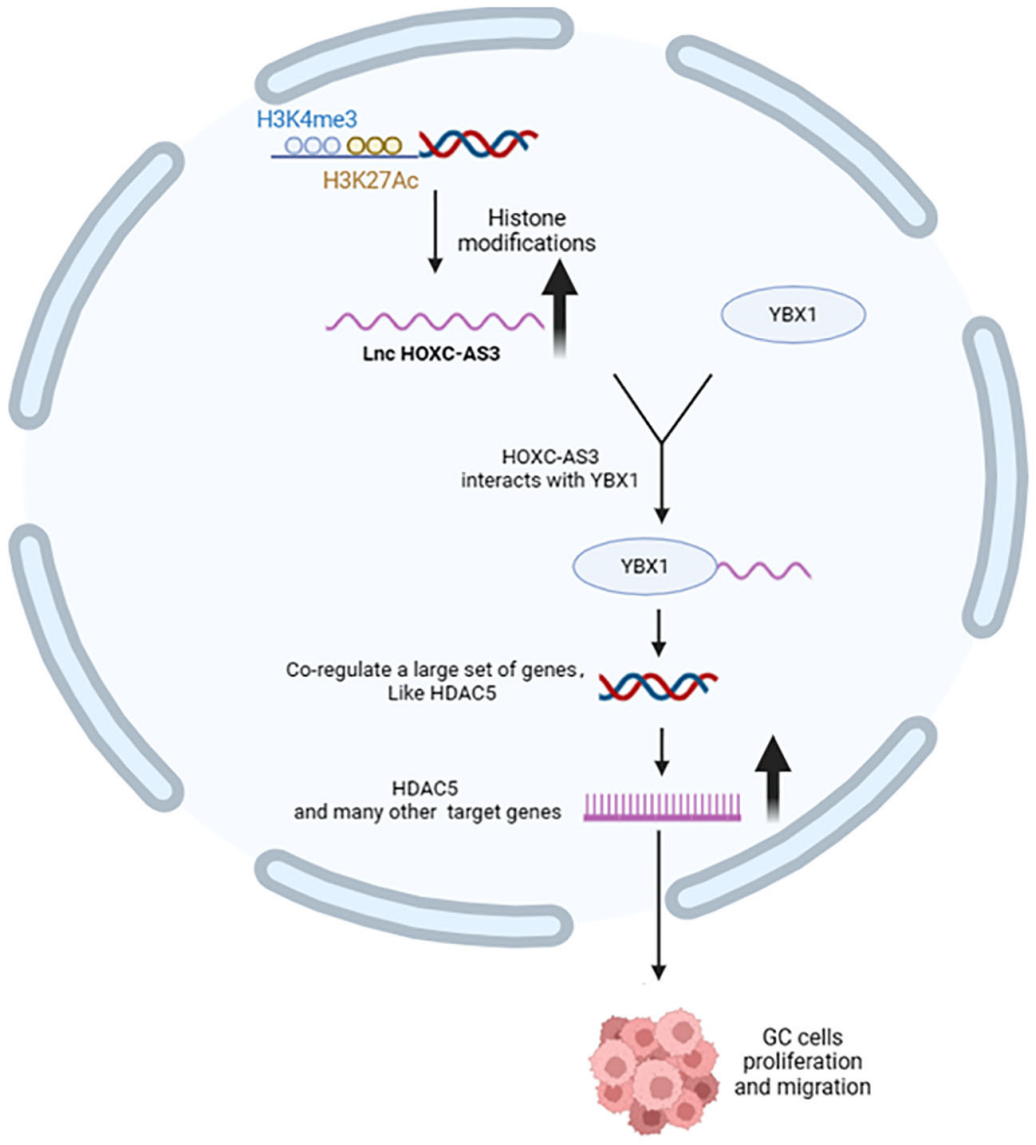
Effects of upstream regulators of YBX1 on its function (lncRNA). HOXC-AS3, activated by H3K4me3 and H3K27ac, interacts with YBX1 to transcriptionally activate various genes, including HDAC5, promoting gastric cancer (GC) cell proliferation and migration.

Long non-coding RNAs (lncRNAs) interact with YBX1, influencing its function and contributing to cancer progression. For example, lncRNA PIN1P1 enhances YBX1 protein expression, promoting gastric cancer via PIN1 upregulation (58). Similarly, HOXC-AS3 lncRNA mediates gastric cancer by binding to YBX1 (**Figure 3**) and regulating cell proliferation and migration (59). RP11-296E3.2 directly binds to YBX1, promoting colorectal cancer proliferation and metastasis through STAT3 activation (60). LncKCND1 binds to YBX1, upregulating YBX1 expression and inhibiting cardiomyocyte hypertrophy (61). DSCAM-AS1 forms a positive feedback loop with YBX1, activating FOXA1 transcriptional network to drive cancer progression (62). RAD51-AS1 interacts with YBX1, improving osteoporosis by regulating osteogenesis (63). LINC01133 binding to YBX1 partially reverses its inhibition on nasopharyngeal carcinoma cell proliferation, migration, and invasion (22). BASP1-AS1 recruits YBX1 to NOTCH3 promoter, driving melanoma development (64). HOXC-AS3 lncRNA regulates breast cancer by binding to YBX1 and activating TK1 transcription (65). MILIP promotes clear cell renal cell carcinoma metastasis by interacting with YBX1 (66). GAS5 binds to YBX1, enhancing G1 cell cycle arrest in gastric cancer (67). HIF1A-AS3 interacts with YBX1, promoting ovarian cancer tumorigenesis by inhibiting p21 and AJAP1 transcription (68). LINC00312 directly binds to YBX1, inducing lung cancer cell migration, invasion, and angiogenesis (69). LINC00472 inhibits lung adenocarcinoma migration and invasion by binding to YBX1 and regulating cell stiffness (70). AC073352.1 lncRNA promotes breast cancer metastasis by stabilizing YBX1 protein and promoting angiogenesis (71). LncRNA HUMT recruits YBX1 to form a new transcriptional complex and activates the expression of forkhead box k1 (FOXK1) in triple-negative breast cancer, thereby promoting lymphangiogenesis and metastasis (72). TCLlnc1 interacts with YBX1 and HNRNPD, promoting peripheral T-cell lymphoma progression (73). CASC11 binds to YBX1, inhibiting p53 pathway and promoting prostate cancer progression (74). PIK3CD-AS2 promotes lung adenocarcinoma progression via YBX1-mediated inhibition of p53 pathway (75). TMEM92-AS1 binds to YBX1, promoting gastric cancer progression through CCL5 upregulation (76). HOTAIR specifically binds to YBX1, promoting PI3K/Akt and ERK/RSK signaling pathways in cancer cells (77). AATBC activates YAP1/Hippo signaling pathway by interacting with YBX1, promoting breast cancer migration and invasion (78). EPB41L4A-AS2 binds to YBX1, enhancing E-cadherin expression and inhibiting EMT progression in NPC (79). LINC00857 interacts with YBX1, regulating apoptosis and autophagy in cancer cells (80). DARS1-AS1 interacts with YBX1, promoting glioblastoma tumorigenesis and radiation resistance (81). lncRNA-AWPPH interacts with YBX1, promoting SNAIL1 translational activation and hepatocellular carcinoma progression (82). LINC00665 binds to YBX1, activating Wnt3a/β-catenin signaling and promoting gastric cancer proliferation and metastasis (83). MIR22HG binds and stabilizes YBX1 protein, affecting cell survival and death signals in cancer cells (84). These interactions highlight the diverse mechanisms through which lncRNAs modulate YBX1 function, making them promising targets for cancer treatment and prognosis evaluation.

Long non-coding RNAs (lncRNAs) exert diverse effects on YBX1 function through epigenetic modification, transcriptional regulation, and other mechanisms, impacting the development and metastasis of various cancers. For instance, lncRNA FOXD3-AS1 enhances YBX1 transcription via H3K27Ac modification, promoting nasopharyngeal carcinoma progression (85). HOXC-AS3 lncRNA binds to YBX1, inhibiting MDM2-mediated YBX1 ubiquitination, and promoting non-small cell lung cancer growth and metastasis (86). lnc-SOX9–4 inhibits YBX1 polyubiquitination and degradation, promoting colorectal cancer progression (87). USP2-AS1 enhances YBX1-HIF1α mRNA binding under hypoxia, increasing HIF1α protein levels and hepatocellular carcinoma growth (88). LNCAROD stabilizes YBX1 via m6A methylation, promoting head and neck squamous cell carcinoma progression (89). RP11-162G10.5 recruits YBX1 to activate GLO1 transcription, regulating breast cancer progression (90). Aerrie lncRNA regulates DNA damage repair via YBX1, maintaining endothelial cell function (91). Linc00312 mediates oral mucosal myofibroblast trans-differentiation through YBX1 (92). SNHG6 enhances YBX1-mediated HIF1α translation, promoting clear cell renal cell carcinoma carcinogenesis (93). MELTF-AS1 directly binds and drives the phase separation of the RNA-binding protein YBX1, which is involved in tumorigenesis, thereby activating ANXA8 transcription and promoting non-small cell lung cancer development (94). Silencing of ENST00000430471 reduces YBX1 mRNA and protein expression (95). Overall, lncRNAs modulate YBX1 activity in tumor cells through various mechanisms, influencing tumor growth, metastasis, and treatment response.

In summary, research has elucidated that long non-coding RNAs (lncRNAs) impact YBX1 function through various mechanisms, including regulating the miRNA/YBX1 signaling axis, interacting with YBX1, epigenetic modification, transcriptional regulation, and more. However, translating these findings into clinically effective therapeutic strategies poses a significant challenge. Efforts to overcome this challenge will require further investigation and development of innovative therapeutic approaches.

### 4.3 CircRNAs and other non-coding RNAs

**Figure 4:**
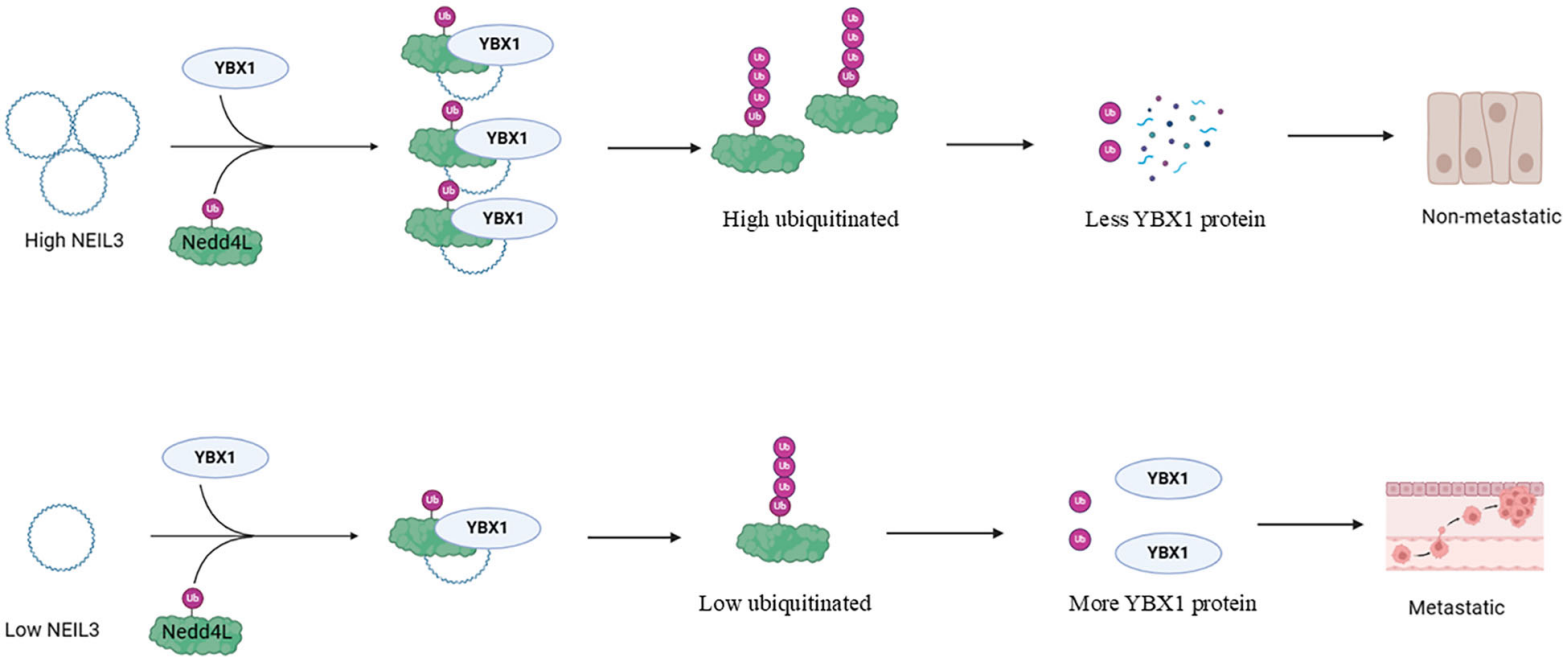
Effects of upstream regulators of YBX1 on its function (CircRNAs and other non-coding RNAs). In non-metastatic tumors, circNEIL3 recruits E3 ligases Nedd4L and YBX1, leading to YBX1 polyubiquitination and subsequent proteasomal degradation. Conversely, in metastatic tumors, circNEIL3 downregulation, in response to specific stresses like TGFβ stimulation, increases YBX1 protein levels, promoting tumor metastasis.

CircRNA represents a distinct class of non-coding RNA molecules characterized by a closed-loop structure, making them resistant to RNA exonuclease degradation and more stable in expression compared to linear RNA. tRNA-derived fragments (TRFs) arise from specific cleavage of mature or precursor tRNA at various sites, constituting a group of small non-coding RNA molecules prevalent in both prokaryotic and eukaryotic transcriptomes. Satellite RNAs (SCS) are another group of small non-coding RNAs typically ranging from 200 to 1500 nucleotides in length and generally lacking protein-coding capacity. circNEIL3 functions by recruiting the E3 ubiquitin ligase Nedd4L to degrade YBX1 (**Figure 4**), thereby inhibiting tumor metastasis (96). Similarly, circRNA-SORE binds to YBX1 in the cytoplasm, preventing its interaction with the E3 ubiquitin ligase PRP19, thus thwarting PRP19-mediated YBX1 degradation and stabilizing YBX1 levels (97). By enhancing liquid-liquid phase separation of YBX1, circASH2 accelerates the decay of TPM4 transcript, impeding liver cancer metastasis (98). CircACTN4 interacts with YBX1 to transcriptionally activate FZD7, promoting the proliferation and metastasis of intrahepatic cholangiocarcinoma (99). Additionally, circIPO7 facilitates YBX1 nuclear translocation and promotes nasopharyngeal carcinoma metastasis and cisplatin chemotherapy resistance by promoting its serine 102 phosphorylation via AKT kinase (100). By inhibiting the phosphorylation of YBX1, cFAM210A suppresses its transactivation function on MET (101). Moreover, CircRABL2B interacts with YBX1 to inhibit MUC5AC, thereby regulating lung cancer stemness and drug sensitivity (102). Furthermore, circ-SAR1A up-regulates YBX1 expression by binding to miR-382, facilitating the growth and invasion of renal cancer cells (103), while hsa_circ_0008035 participates in gastric cancer occurrence by targeting the miR-375/YBX1 axis (104). CircFAT1 (e2) interacts with YBX1 to suppress the progression of gastric cancer by targeting miR-548g (105). Additionally, endogenous TRFs interfere with the stability of various oncogenic transcripts in breast cancer cells by replacing 3’ untranslated regions (UTRs) on YBX1 (106). Satellite RNA inhibits YBX1 nuclear translocation under stress conditions, reducing its DNA damage repair function (107). RNA-m5C methylation mediated by NSUN2/YBX1/QSOX1 confers resistance to treatment in non-small cell lung cancer (108). piR-YBX1, an RNA that interacts with PIWI, down-regulates YBX1 expression by directly binding to YBX1 mRNA and promoting its degradation. In addition, the overexpression of piR-YBX1 can inhibit the binding of YBX1 to RAF1, a key molecule in the MAPK signaling pathway, and then inhibit p-MEK and p-ERK1/2, and inhibit the proliferation and metastasis of triple-negative breast cancer (109). In summary, various non-coding RNA molecules, including circRNA, TRFs, and satellite RNA, modulate YBX1 protein activity and stability, thereby influencing tumor metastasis and drug sensitivity. These molecules play pivotal roles in tumor biology by interacting with YBX1, impacting its phosphorylation, nuclear translocation, transcriptional activity, and miRNA interaction.

### 4.4 Transcription factors or other genes

Transcription factors are essential proteins that bind to specific DNA sequences, regulating gene expression within cells. Fibrillation (FBL) protein, for instance, increases YBX1 binding to the BRCA1 promoter in breast cancer, thereby promoting BRCA1 expression (110). CTPS1 contributes to the malignant progression of triple-negative breast cancer by transcriptionally activating YBX1 (111). C1QBP regulates YBX1 and suppresses androgen receptor (AR)-enhanced invasion of renal cell carcinoma cells (112). In lung squamous cell carcinoma, gasdermin E (GSDME) interacts with YBX1, facilitating its transport to the nucleus, where it directly promotes mucin expression (113). THOC3 interaction with YBX1 modifies PFKFB4 mRNA, promoting the progression of lung squamous cell carcinoma (114). In glioma stem cells, PLK1 interacts with YBX1 and phosphorylates serine residues YBX1–176 and 174, leading to reduced YBX1 levels and inhibition of its nuclear translocation, inducing apoptosis and DNA damage in glioma stem cells (115). In colorectal cancer, PRMT5 mediates YBX1 methylation, regulating NF-kappa B activity (116). hTERT enhances colorectal cancer proliferation and migration by recruiting YBX1 and increasing NRF2 expression (117). In prostate cancer, Aurora kinase-A (AURKA) collaborates with YBX1 to promote aggressive carcinogenic phenotypes and chemoresistance (118). In hepatocellular carcinoma, IRGM enhances the interaction between YBX1 and S6K1 kinase, increasing YBX1 phosphorylation and nuclear localization, and enhancing PD-L1 transcription (119). CDH1 downregulation in non-small cell lung cancer promotes EGFR transcription by activating YBX1 phosphorylation (120). In leukemia, GAS6 AS1 binds directly to YBX1, promoting its interaction with MYC and resulting in the activation of MYC target genes associated with leukemia progression (121).

### 4.5 Drugs or small molecule inhibitors

**Figure 5:**
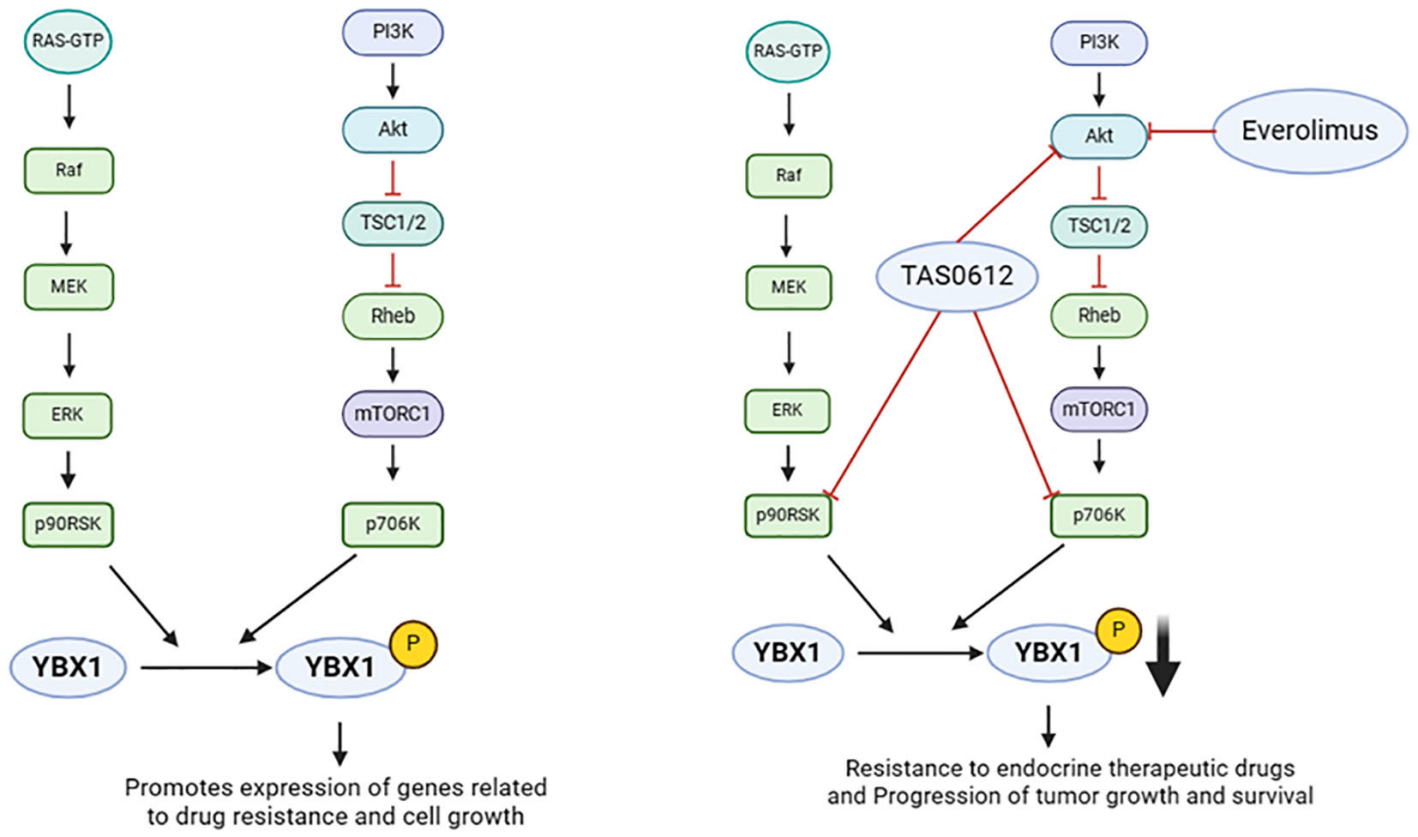
Effects of upstream regulators of YBX1 on its function (Drugs or small molecule inhibitors). YBX1 phosphorylation at ser102 by AKT, p70S6K, and p90RSK promotes its nuclear translocation. Phosphorylated YBX1 (pYBX1) enhances the expression of drug resistance and cell growth-related genes. TAS0612 and everolimus target YBX1 phosphorylation to address anti-estrogen resistance and advanced breast cancer.

Drugs and small molecule inhibitors are pivotal in cancer therapy, capable of disrupting tumor cell signaling, proliferation, migration, and drug resistance through specific molecular targets. In the realm of YBX1 functional studies, a range of drugs and inhibitors have been identified to impact YBX1 activity and its regulatory network, offering a novel approach to cancer treatment. For instance, sunitinib, a small molecule inhibitor, reduces ectopic endometrial cell migration via the p-VEGFR-PI3K-AKT-YBX1-Snail signaling pathway, presenting promise as a targeted therapy (122). TAS0612 and everolimus target YBX1 phosphorylation (**Figure 5**), addressing anti-estrogen treatment resistance in advanced breast cancer (123). Interactions between C1QBP and YBX1 result in decreased C1QBP levels, enhancing YBX1 phosphorylation and nuclear translocation in renal cell carcinoma (124). YBX1 mediates MIA/CD-RAP-dependent p54^nrb^ (non-POU-domain-containing octamer-binding protein) transcriptional activation, with the p54^nrb^ promoter serving as a MIA/CD-RAP-mediated regulator implicated in chondrogenesis and malignant melanoma progression (125). Melanoma inhibitory active protein (MIA) further fuels melanoma progression by activating YBX1 (126). SIAH1 as new E3 ligase, through the ubiquitin of YBX1 reversal of epithelial ovarian cancer chemotherapy drug resistance (127). Additionally, the 5’-untranslated region of the melanoma tumor suppressor p16INK4a acts as a cellular IRES, controlling mRNA translation under hypoxia via YBX1 binding (128). The research conducted by Dr. Sanjay V. Malhotra revealed the YBX1 inhibitors like SU056 provide valuable insights into the mechanisms of YBX1’s oncogenic functions. For instance, YBX1 inhibition can lead to the downregulation of spliceosome pathway components and the upregulation of apoptosis and RNA degradation pathways. This mechanistic understanding helps in the rational design of combination therapies where YBX1 inhibitors can be used alongside traditional chemotherapeutic agents to enhance treatment efficacy and overcome drug resistance (129). In summation, intervening in YBX1 function through drug and small molecule inhibitors presents novel strategies for treating various cancers. These studies deepen our understanding of YBX1’s role in cancer development and provide a crucial scientific foundation for future drug development in cancer treatment.

## 5 The relationship between YBX1 and disease efficacy and prognosis

In addition to its roles in tumor progression and other biological processes, YBX1 is also emerging as a key player in immune regulation. It has been established that YBX1 functions as a regulator of immune responses, including the expression of cytokines such as IL-2, IL-6, and the signaling of NF-κB in immune cells. In renal cancer, Wang et al. confirmed that YBX1 interacts with G3BP1 to upregulate its downstream target SPP1, activating the NF-κB signaling pathway and promoting metastasis of renal cancer cells (130). In gastric cancer, YBX1 upregulates the expression of SPP1, promoting gastric tumor-initiating cells through the ITGB1/YBX1/SPP1/NF-κB signaling pathway (131). In cholangiocarcinoma, KIF14 binds to the G3BP1/YBX1 complex, enhancing their interaction and leading to increased NF-κB promoter activity. This activation of the NF-κB pathway promotes tumor proliferation, lymphatic metastasis, and chemotherapy resistance (132). CD4^+^ T cells in renal cell carcinoma stimulate cancer cell proliferation by activating the YBX1/HIF2α signaling pathway (133). These findings underscore the importance of YBX1 in immune regulation and highlight new avenues for therapeutic intervention in immune-related disorders.

As a multifunctional RNA-binding protein, YBX1 plays crucial roles in the onset, progression, treatment efficacy, and prognosis of various diseases. Elevated YBX1 expression is linked to poorer prognosis in nasopharyngeal carcinoma (NPC), suggesting its potential as a prognostic biomarker for NPC patients, aiding clinicians in treatment evaluation and strategy adjustment (134). In acute myeloid leukemia patients, increased YBX1 expression correlates with adverse genomic abnormalities, indicating its relevance to disease pathogenesis (135). Moreover, heightened YBX1 levels are significantly associated with tumor differentiation, size, and lymph node metastasis in solid tumor patients, underscoring its role in tumor progression (136). In oral squamous cell carcinoma (OSCC), YBX1 and RAN are essential for cell proliferation and IL-4 expression, with their overexpression linked to poor prognosis, suggesting their involvement in OSCC growth and immune response regulation (137). Furthermore, YBX1 and ESR1 serve as biomarkers for adverse outcomes in breast cancer patients (138). Analyses of YBX1 interaction networks reveal potential therapeutic targets for adenocarcinoma, while exploring PABPC1, an YBX1 interaction partner, offers avenues for discovering new drug targets in lung adenocarcinoma (139, 140). These findings underscore the multifaceted roles of YBX1 in tumor biology and provide novel strategies and targets for advancing cancer therapy.

## 6 Problems and prospects

In the realm of YBX1 research, despite significant advancements, numerous questions remain unanswered. While it is established that YBX1 participates in diverse cellular functions, the precise mechanisms governing its roles in transcription, translation, and DNA damage repair require further elucidation. Given its pivotal involvement in tumor progression, growth, heart and brain diseases, bone differentiation, chondrogenesis, and adipogenesis, a pressing task is confirming these mechanisms *in vivo*. Looking ahead, the future research trajectory and prospects for YBX1 entail the development of small molecule drugs or inhibitors targeting YBX1, offering a promising new avenue for cancer treatment strategies.
